# VSD——治疗手术感染切口的好思路

**DOI:** 10.3779/j.issn.1009-3419.2018.04.26

**Published:** 2018-04-20

**Authors:** 朝阳 梁

**Affiliations:** 中日友好医院胸外科 Department of Thoracic Surgery, China-Japanese Friendship Hospital

外科手术后伤口感染是外科手术治疗最常见的并发症之一，尤其对于文中涉及的食管癌根治术及脓胸纤维板剥脱术而言，Ⅱ-Ⅲ类切口发生术后感染的几率更高，处理相对棘手。传统治疗措施的关键在于充分引流和加强换药，在此基础上衍生出众多旨在促进局部肉芽组织生长、减少伤口渗出的"偏方"，如浓钠、高糖等等，尚缺乏确凿的研究证据支持。

负压封闭引流技术（vacuum sealing drainage, VSD）较早应用于四肢开放性骨折复位内固定、脊柱手术、大面积烧伤或软组织损伤等的治疗中，其共同特点在于存在较高的感染风险，同时需最大限度避免因感染造成的严重并发症（内固定物取出、脓毒血症等）。因临床疗效肯定，VSD的适应症逐渐扩大到腹部手术，尤其是腹壁较厚的患者，负压吸引有利于降低切口张力，同时消除软组织间隙，降低切口脂肪液化等的风险^[1]^。此外还有大量关于VSD联合药物治疗感染及VSD应用于动物咬伤治疗的报道。

关于VSD在胸部手术中的应用，尤其是对感染伤口的治疗效果报道有限，仅有少量联合应用VSD和转移肌瓣治疗胸骨正中切口术后胸骨骨髓炎的报道^[2]^。本文提出将VSD应用于食管术后吻合口瘘或慢性脓胸导致的伤口感染，获得满意疗效，有一定的借鉴意义。探究VSD的优势，从宏观上讲，利用合适的负压，将脓液自感染创面“吸出”，能起到充分引流的作用，利用负压消灭死腔的同时亦能促进软组织粘连与愈合；微观机制有待进一步研究，改善局部缺血-再灌注损伤是可能的机制之一，亦可能通过调控VEGF和miRNA表达、增加细胞抗氧化应激反应、抑制活性氧自由基和炎症介质释放等发挥效应。

VSD的应用同样存在局限。实际操作中最大的挑战在于维持负压，胸壁、腹壁一般能提供足够平整的“使用面积”，但对于三切口食管癌根治术颈部切口、后外侧切口后缘等易出现伤口感染的特殊部位，或当存在引流管遮蔽等因素时，如何密闭伤口并维持负压，对医疗、护理提出较高的要求，或限制该应用推广。对食管癌术后吻合口瘘病例而言，过大的负压是否会延迟瘘口闭合，存在疑虑，需要积累病例增加临床经验。对于慢性脓胸行纤维板剥脱的患者，术中常见损伤脏层胸膜致肺漏气，故术后经常应用负压胸引，若肺复张不满意再次出现脓胸继发伤口感染，仅靠VSD负压恐难以维持。因此，据术中情况选择合适病例应用VSD治疗十分重要。如果再考虑到成本-收效比，则VSD的适用范围更需仔细讨论。最后还需强调的是，VSD治疗只是感染伤口治疗的组成部分之一，包括积极营养支持、合理抗生素选用在内的全身治疗同样非常重要。

未来，可以通过临床经验积累或设计实施临床研究，制定用于预防、治疗胸部手术术后感染的VSD使用适应证和压力控制范围，进一步探索其治疗疗效，有效规避副反应。对合适的患者而言，VSD可能成为实践个体化治疗的理想选择。
